# Stabilized Lignin Nanoparticles for Versatile Hybrid and Functional Nanomaterials

**DOI:** 10.1021/acs.biomac.2c00840

**Published:** 2022-10-14

**Authors:** Mohammad Morsali, Adrian Moreno, Andriana Loukovitou, Ievgen Pylypchuk, Mika H. Sipponen

**Affiliations:** Department of Materials and Environmental Chemistry, Stockholm University, Svante Arrhenius väg 16C, SE-106 91Stockholm, Sweden

## Abstract

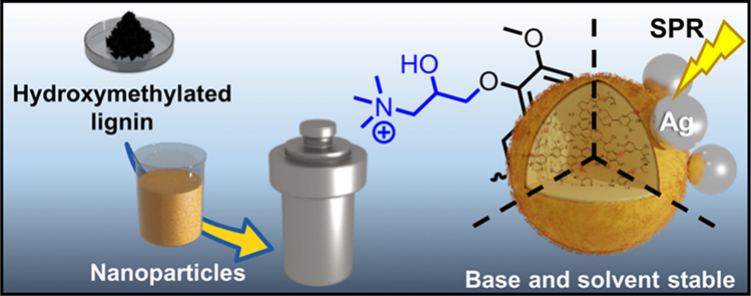

Spherical lignin nanoparticles are emerging biobased nanomaterials, but instability and dissolution in organic solvents and aqueous alkali restrict their applicability. Here, we report the synthesis of hydroxymethylated lignin nanoparticles and their hydrothermal curing to stabilize the particles by internal cross-linking reactions. These colloidally stable particles contain a high biobased content of 97% with a tunable particle size distribution and structural stability in aqueous media (pH 3 to 12) and organic solvents such as acetone, ethanol, dimethylformamide, and tetrahydrofuran. We demonstrate that the free phenolic hydroxyl groups that are preserved in the cured particles function as efficient reducing sites for silver ions, giving rise to hybrid lignin–silver nanoparticles that can be used for quick and facile sensing of hydrogen peroxide. The stabilized lignin particles can also be directly modified using base-catalyzed reactions such as the ring-opening of cationic epoxides that render the particles with pH-dependent agglomeration and redispersion properties. Combining scalable synthesis, solvent stability, and reusability, this new class of lignin nanoparticles shows potential for its use in circular biobased nanomaterials.

## 1 Introduction

Nanoparticles and hydrocolloids have become essential ingredients in many technical, food, and life science applications. In particular, biobased nanoparticles are emerging as multifunctional alternatives to fossil-derived and conventional nanoparticles. In this context, the advent of lignin hydrocolloids as biobased building blocks has gathered considerable attention and has transformed the possibilities of using lignin in many prospective end-uses.^1^ Lignin is a plant-based polyphenol that allows trees to have a strong structure and protection against pests and microorganisms.^2−4^ Considering its inherent attractive properties such as high carbon content (>60 atom %) and thermal stability, biodegradability, antioxidant activity, and the absorbance of UV irradiation,^5−7^ lignin has emerged as a prime candidate for biobased nanoparticles and nanohybrids.^8−11^ In particular, the spherical and colloidally stable lignin nanoparticles (LNPs) display benefits compared to crude lignin powders that are poorly soluble in many common solvents and are heterogeneous both in molecular weight and distribution of functional groups.^12−15^

In addition to its inherent functionality, covalent and noncovalent modifications of lignin can extend the applicability of LNPs.^1^ For instance, Zhao et al. aminated lignin by the Mannich reaction prior to the particle formation and demonstrated the corresponding LNPs for the conjugation of histidine for pH-responsive drug release in cancer treatment.^16^ An alternate strategy is to modify the surfaces of LNPs in the colloidal state by adsorption of water-soluble polymers^2,13^ and proteins^17^ including enzymes.^18−22^ However, the solubility of LNPs in organic solvents and their preferred production as aqueous dispersions restrict the use of other common nanoparticle modification techniques that have been used extensively since the discovery of nanoscience.^1^ In fact, the vast majority of covalent functionalizations that can be readily used in the solution state^23^ cannot be conducted in aqueous dispersions due to the predominant side reactions such as hydrolysis during esterification.

It has become apparent that overcoming the solvent instability of LNPs would render them plausible alternatives to fossil-based and inorganic nanoparticles such as polystyrene or silica nanoparticles. This can be achieved via internal cross-linking of LNPs by addition of a cross-linker during their supramolecular assembly.^24−27^ Another means to stabilize LNPs is by enzymatic treatment with oxidoreductive enzymes such as laccases.^28,29^ Wang et al. solvent-fractionated birchwood soda lignin sequentially with isopropanol, ethanol, and methanol and treated the methanol-soluble fraction with laccase at pH 10.^29^ The product was isolated and used to assemble LNPs for reduction of silver ions under alkaline conditions. The hybrid silver–lignin particles were thereafter used in photocurable hydrogel formulations. However, long-term stability of the LNPs under alkaline conditions or in anhydrous organic solvents was not reported.

Regardless of the modification route, one of the shortcomings of covalent cross-linking schemes is the consumption of aliphatic and phenolic hydroxyl groups that are the most important sites for functionalization and biodegradation of lignin. In fact, the free phenolic hydroxyl groups are the prime functionalities defining biodegradability, redox activity, and antimicrobial activity of lignin.^30−33^ To overcome this hurdle, Moreno et al. prepared nanoparticles using lignin oleate as a precursor, leading to sufficiently long stability under acidic (pH 2) and alkaline (pH 12) conditions to perform surface-specific modifications without any cross-linking.^34^ These authors speculated that the partial esterification with natural fatty acids would not impede the biodegradability of lignin. However, all of these previous approaches have some shortcomings that restrict the scalability and ability to carry out atom-efficient functionalization in the dispersion state. In particular, any covalent modification process should aim at a high mass yield and biobased content and minimize the generation of wastewater.

There is thus an urgent need for robust and readily scalable methods for the production of stabilized LNPs, with sufficient control over the particle size and concentration, while preserving the inherent hydroxyl groups that enable dispersion state functionalization toward advanced biobased materials. Here, we report the preparation of internally cross-linked nanoparticles from hydroxymethylated lignin at a high overall yield and biobased content. The hydroxymethylated lignin nanoparticles (HLNPs) preserve the free phenolic hydroxyl groups, while the new aliphatic hydroxyls increase the solubility of lignin in aqueous organic solvents and provide a means to control the particle size and increase the concentration of the produced hydrocolloids. Additionally, the hydroxymethyl groups can be used as active sites for further modification or cross-linking of lignin. We show that hydrothermally cured HLNPs are stable in organic solvents and aqueous media over a broad pH range for several weeks, which opens up a new possibility of using them as templates for lignin–metal and lignin–inorganic hybrid nanoparticles. By exploiting the preserved phenolic hydroxyl groups, we demonstrate the formation of colloidally stable hybrid silver–lignin nanoparticles as colloidal sensors for reactive oxygen species using hydrogen peroxide as an example. We further present covalent surface functionalization of the internally cross-linked particles through a base-catalyzed epoxy ring-opening reaction to obtain pH-responsive hydrocolloids of broad interest for different applications.

## 2 Experimental Section

### 2.1 Materials

Softwood kraft lignin (SKL, BioPiva 100 pine kraft lignin), sodium hydroxide (VWR), formalin solution (formaldehyde, 37%, Sigma-Aldrich), glycidyltrimethylammonium chloride (GTMA, Sigma-Aldrich), silver nitrate (Merck), ammonia solution (35%, Thermo Fisher Scientific), hydrochloric acid (35%, VWR), acetone (Honeywell), and hydrogen peroxide (H_2_O_2_, 30%, Merck) were used as received.

### 2.2 Preparation of Hydroxymethylated Lignin

In a round bottom glass, 11.25 mL of aqueous sodium hydroxide (1 M, 11.25 mmol) was added to 5 g of kraft lignin (dry basis) and the mixture was allowed to stir at room temperature. Next, 8.75 mL of deionized water was added to the stirring mixture and the temperature was increased to either 60 or 85 °C. Then, 5 mL of formalin solution (37% formaldehyde, 66 mmol) was added dropwise to the lignin solution. The reaction was allowed to take place for 3 h. Next, the product solution was quenched in cold deionized water and adjusted to pH 3 with drop by drop addition of 0.1 M hydrochloric acid. The precipitate was centrifuged and washed three times with deionized water to remove extra acid and reach pH 3.2. The purified products were freeze-dried to collect dry hydroxymethylated lignin, with 89% reaction yield (4.45 g) at 60 °C and 90% reaction yield (4.50 g) at 85 °C.

### 2.3 Preparation of Nanoparticles from Hydroxymethylated Lignin

Colloidal nanoparticles from hydroxymethylated lignin were prepared following a literature method. Briefly, 0.5 g of dry hydroxymethylated lignin was dissolved in a solvent mixture of 30 g of acetone and 10 g of deionized water with stirring for 3 h at room temperature. The solution was vacuum-filtered by passing it through a 0.45 μm pore size glass fiber filter to remove any insoluble solids. Finally, 120 g of deionized water was added to the stirring solution to form a colloidal dispersion of hydroxymethylated lignin. Acetone was removed using a rotary evaporator under a reduced pressure at 40 °C.

### 2.4 Cross-Linking of HLNPs Using the Hydrothermal Process

A total of 20 mL of HLNP colloidal dispersion (5 g L^–1^) was hydrothermally cured in a 40 mL Teflon-sealed reactor at 150 °C overnight. Please note that Teflon reactors should be clean from any dust or dirt particles to avoid interparticle cross-linking and aggregation during the hydrothermal curing step.

### 2.5 Direct Modification of HLNPs Using GTMA

A dispersion of HL60NP (10 mL, particle concentration 1 g L^–1^) was reacted at pH 12 (1 M NaOH) with 40 μL of GTMA for 2 h at 80 °C. The reacted particles were dialyzed in deionized water for 24 h. The dialyzed product was in the form of a precipitate that could be redispersed by increasing or decreasing the pH.

### 2.6 Assembly of Silver Nanoparticles on Cross-Linked Lignin Nanoparticles

Silver nanoparticle-modified HLNPs were synthesized using silver ammonia solution (Tollens’ reagent) as the precursor.^35^ Next, 10 mg of silver nitrate (AgNO_3_) was dissolved in 3 mL of deionized water and 40 μL of aqueous ammonia solution was added and stirred to dissolve all the brown precipitate formed. Then, 1 mL of HLNP dispersion (10 mg/mL, concentrated using centrifugation) was added dropwise to the stirring silver ammonia solution and was further stirred for 2 h. After formation of metallic silver, samples were purified by repeated steps of centrifugation and redispersion in deionized water.

For the UV–vis kinetic experiments, the concentration of the silver ammonia solution was one half of the aforementioned procedure. HLNP dispersion (20 μL, 5 mg/mL) was added to the silver ammonia solution, and the absorbance spectra were collected every 5 min for 120 min to follow the reaction kinetics.

### 2.7 Colorimetric Detection of H_2_O_2_

For the detection of H_2_O_2_, 10 μL of Ag-HLNP dispersion was diluted using phosphate buffer (20 mM, pH 7.4) to reach an absorbance of less than 1. Then, different amounts of dilute 20 mM H_2_O_2_ solution were used to measure the absorbance change in different concentrations. The final volume of the solution was controlled by the addition of deionized water. UV–vis spectra of samples with different concentrations of H_2_O_2_ were measured after 30 min.

### 2.8 Nuclear Magnetic Resonance (NMR) Spectroscopy

Quantitative analysis of hydroxyl groups of SKL and hydroxymethylated lignin was conducted using ^31^P NMR spectroscopy,^36^ following the adopted procedure previously reported by us.^37^ Briefly, the dry lignin or hydroxymethylated lignin sample (30 mg) is phosphitylated with 2-chloro-4,4,5,5-tetramethyl-1,3,2-dioxaphospholane (0.9 mmol) in the presence of *N*-hydroxy-5-norbornene-2,3-dicarboxylic acid imine (0.010 mmol) as an internal standard and chromium(III) acetylacetonate as a relaxation agent. The ^31^P NMR experiments (256 scans, 10 s relaxation delay) were performed with 90° pulse angle and inverse gated proton decoupling.

### 2.9 Dynamic Light Scattering (DLS)

The particle size and zeta potential were measured using a Zetasizer Nano ZS90 instrument (Malvern Instruments Ltd., UK). Zeta potential measurements were measured using a dip cell probe. Measurements were carried out with three replicates of the particle diameter (Z-average, intensity mean) and zeta potential, and the mean values were used for the study.

### 2.10 Differential Scanning Calorimetry (DSC)

DSC measurements were performed on a Netzsch DSC 214 Polyma with N_2_ as the purge gas (60 mL/min) and using a heating rate of 10 °C/min in the 25–300 °C temperature range.

### 2.11 Scanning Electron Microscopy (SEM)

SEM imaging was conducted using a JEOL JSM-7401F (JEOL Ltd., Japan) using a secondary electron detector. Samples for imaging were diluted 50 times using deionized water and drop-cast on a silicon wafer.

### 2.12 Fourier Transform Infrared (FTIR) Spectroscopy

FTIR data were collected using a Varian 610-IR FTIR spectrometer. The infrared absorbance of samples was measured using an attenuated total reflection–Fourier transform infrared spectrometer (ATR-FTIR) in the range of 450–4000 cm^–1^.

## 3 Results and Discussion

### 3.1 Hydroxymethylation of Lignin and Effect of Temperature

**Figure 1:**
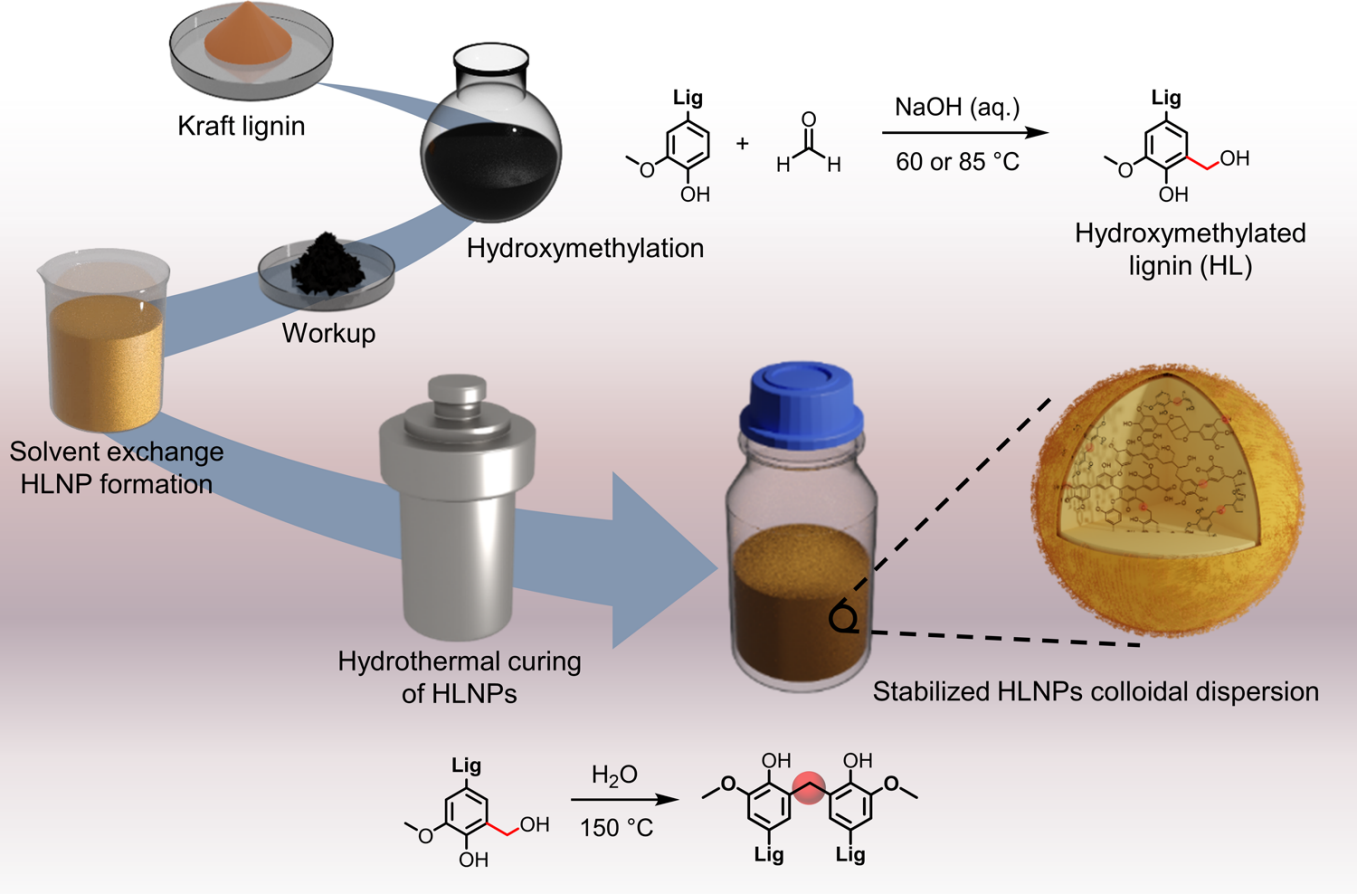
Illustration of the preparation of HLNPs and their hydrothermal curing to produce stabilized HLNPs.

Our approach starts with the preparation of hydroxymethylated lignin (HL) from previously characterized SKL^18^ via electrophilic aromatic substitution using formaldehyde as an electrophile (Figure 1).^3^ Hydroxymethylation of lignin is highly affected and governed by the source of lignin (molecular weight and steric hindrance),^5^ type of catalysts,^38^ temperature,^39,40^ formaldehyde concentration,^41^ and pH.^42,43^ To obtain materials with different extents of reaction at the C5 position of the aromatic ring, we hydroxymethylated SKL at 60 and 85 °C keeping the concentrations of sodium hydroxide and formaldehyde constant. The recovered products of HLs (HL60 and HL85) were thereafter used for the formation of hydrocolloids and later hydrothermally cured to internally cross-link the colloidal particles (Figure 1) and further evaluate their stability under challenging solvent conditions.

**Figure 2:**
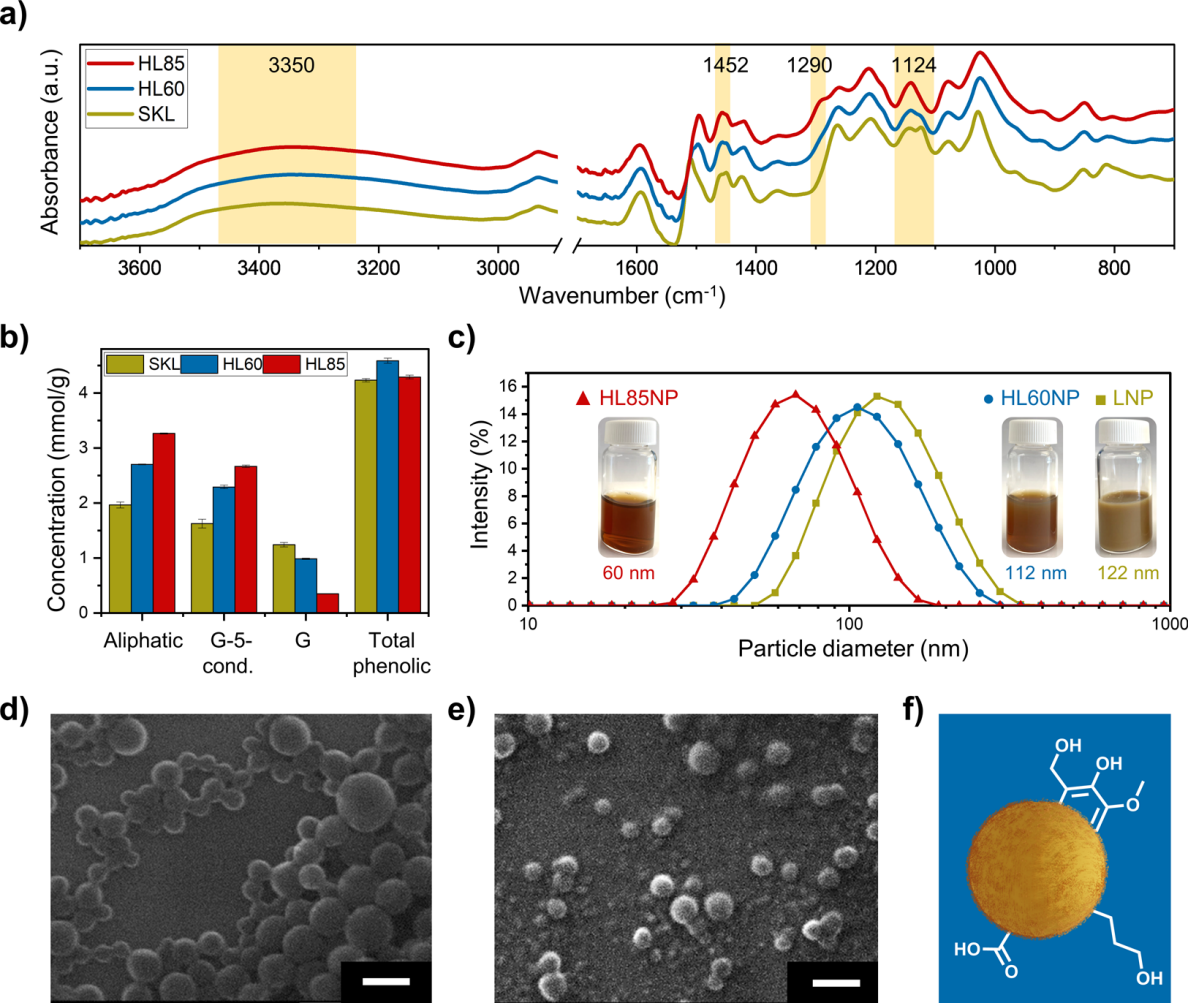
Hydroxymethylation of SKL and the preparation of corresponding LNPs. (a) FTIR spectra of SKL, HL60, and HL85 (lignin was hydroxymethylated at 60 and 85 °C, respectively). (b) Quantitative ^31^P NMR spectroscopy analysis of SKL, HL60, and HL85. (c) Particle size distribution (DLS, hydrodynamic diameter) of LNPs and nanoparticles prepared from HL60 and HL85. SEM images of (d) HL60NPs and (e) HL85NPs. Scale bars correspond to 100 nm. (f) Schematic model of the functional groups present on the surfaces of HL60NPs and HL85NPs.

Successful hydroxymethylation of lignin was analyzed using spectroscopic techniques. As can be seen from the FTIR spectra of different samples (SKL, HL60, and HL85), obvious differences in the absorbance bands of SKL after hydroxymethylation are noticeable (Figure 2a). First, there is an increase in the hydroxyl content of reacted lignin, which can be seen by the increase of the absorbance intensity at 3350 cm^–1^. Second, the disappearance of the band at 1124 cm^–1^, the change in the band at 1452 cm^–1^, and the appearance of a shoulder at 1290 cm^–1^ suggest that a more extensive hydroxymethylation reaction occurred at 85 °C than at 60 °C. These results are in agreement with the literature^39,44−46^ and our quantitative ^31^P NMR data (Table S1). A marked temperature-dependent increase in the content of aliphatic hydroxyl groups in the HLs was determined compared to the parent kraft lignin (Figure 2b). This trend shows an increase in the aliphatic hydroxyl content upon increasing the hydroxymethylation temperature from 60 to 85 °C and matches with the trend of the change of condensed and noncondensed guaiacyl (free 5-position in the aromatic ring) moieties. It is visible that on increasing the reaction temperature in the presence of formaldehyde, the content of noncondensed guaiacyl decreased by 52% in HL85 and 20% in HL60, while that of condensed guaiacyl (carbon–carbon or ether linkage at the 5-position) increased by 64% in HL85 and 41% in HL60. At the same time, the phenolic hydroxyl content remained essentially unchanged compared to the parent lignin. Based on the ^31^P NMR data, the mass fraction of hydroxymethyl groups in HL85 was about 3 wt %.

### 3.2 Preparation of HLNPs

Lignin as an amphiphilic polymer can undergo phase separation and form nanoparticles by aggregation driven by hydrophobic interactions. There have been attempts for synthesizing nanoparticles from HL using, for example, acid precipitation;^47^ however, particles prepared using the solvent shifting technique show a better spherical geometry and colloidal stability.^48^ The particles prepared using solvent shifting from HL60 and HL85 demonstrate excellent colloidal stability but interestingly a smaller size compared to conventional LNPs prepared under identical conditions (Figure 2c). The particle sizes analyzed by DLS show that the final size of particles depends on the degree of hydroxymethylation (Figure 2c). Considering the effect of temperature on the hydroxyl content of HL shown in this study, this trend is in line with the results of the study by Pylypchuk et al. who found that increasing the content of aliphatic hydroxyl groups of lignin can result in smaller particle sizes.^49,50^ It is worth noting that as the particle size decreases, the turbidity in the colloidal dispersion drops, which is the result of smaller angles of light scattering (Figure 2c). The SEM images confirm this trend. Larger particles were observed in the case of HL60NP compared to the more extensively hydroxymethylated HL85NP (Figure 2d,e). Most importantly, these hydroxymethylated particles remain reactive not only due to the hydroxymethyl groups but also owing to the presence of free phenolic hydroxyl groups on their surfaces (Figure 2f). Additionally, HLs show increased solubility in the solvent mixture (acetone/water 3:1 w/w) used in the solvent shifting method, which results in higher concentrations of HLNPs in the final aqueous dispersions. In addition, the particle size of the HLNPs can be tuned by increasing the initial concentration of HL85 beyond that possible with unmodified lignin while still achieving a good mass yield (>80%) without solvent fractionation^12,15^ (Figure S1).

### 3.3 Hydrothermal Curing of HLNPs for Exceptional Colloidal Stability under Challenging Conditions

With colloidally stable hydroxymethylated nanoparticles available, our next step was to explore the possibility to hydrothermally cure the particles in the dispersion state and investigate their stability in organic solvents and as a function of pH in aqueous dispersions. To find a suitable curing temperature, we subjected the HLs to thermal analysis. DSC thermograms recorded from HL60 and HL85 revealed that the degree of hydroxymethylation affects their thermal behavior. The area of the exothermic peak associated with the condensation reaction of HL between 120 and 170 °C was proportionally larger in HL85 compared to HL60 (Figure S2). Successful curing of HL60 and HL85 in the dry state at 150 °C was obvious also by comparing the solubilities in an acetone/water (3:1 w/w) solvent mixture of the original kraft lignin and the two cured samples. In contrast to their parent lignin that was fully solubilized, the HLs showed low solubility that expectedly followed the order HL60 > HL85 (Figure S3).

**Figure 3:**
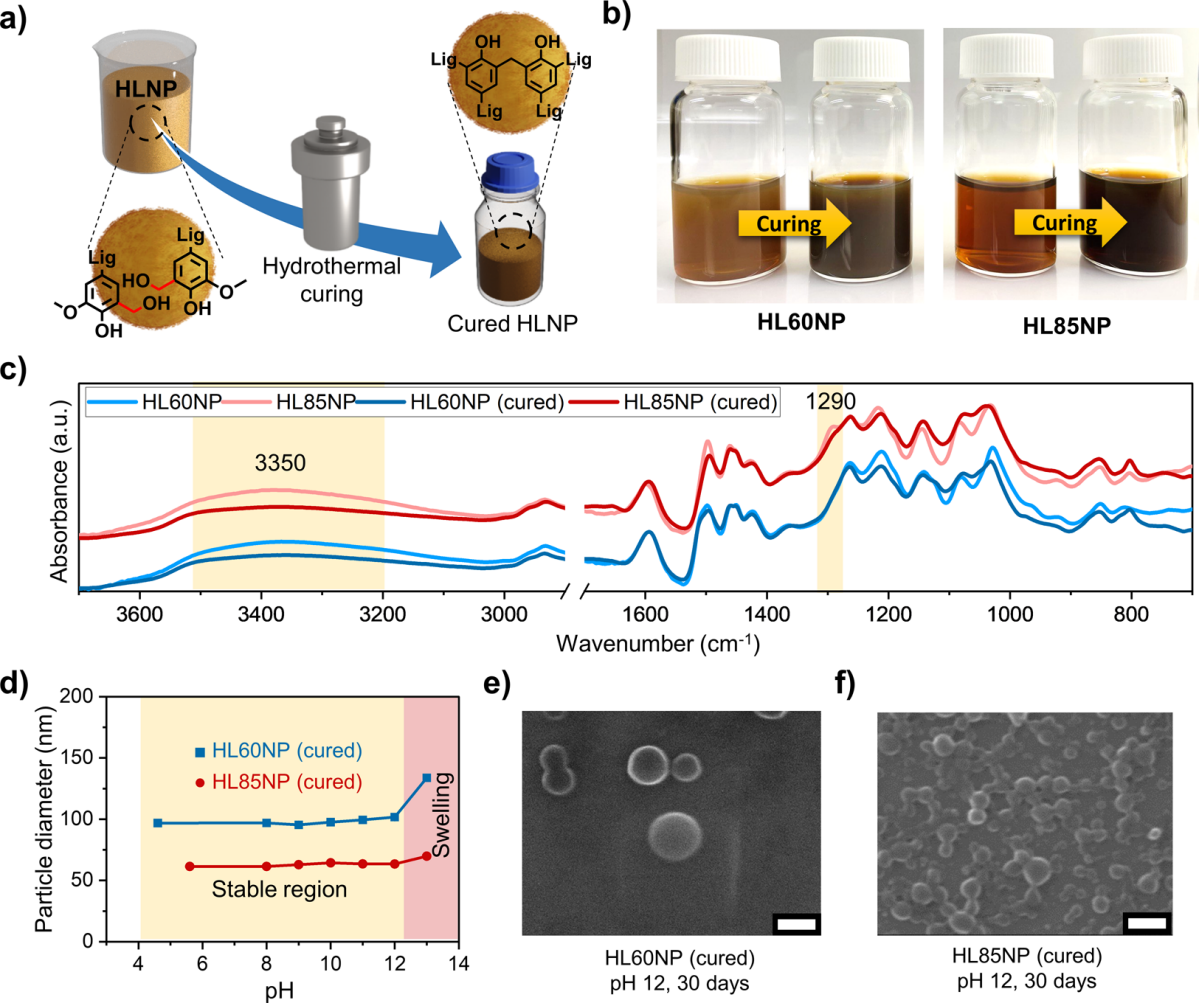
Hydrothermal processing for internal cross-linking of nanoparticles (HLNPs) prepared from lignin hydroxymethylated at 60 and 85 °C (HL60 and HL85). (a) Process for the preparation of cross-linked HLNPs (HL60NP and HL85NP) from their colloidal dispersions. (b) Digital images of HLNPs before and after hydrothermal processing. (c) ATR-FTIR spectra of cross-linked HLNPs and the comparison between their spectra before and after hydrothermal processing. (d) Particle size of cross-linked HLNPs in aqueous media with different pH values after 20 days. SEM images of cross-linked particles conditioned at pH 12 demonstrating their structural stability: (e) HL60NP and (f) HL85NP. Scale bars correspond to 100 nm.

Colloidal stability under different challenging conditions is essential for many applications of nanoparticles.^51^ To achieve this, HLNPs originating from HL60 and HL85 were subjected to hydrothermal curing at 150 °C (Figure 3a). The resulting particles were colloidally stable at room temperature without any sign of precipitation or aggregation for at least 6 months (Figure 3b). The cured HLNPs were dried and studied using FTIR to reveal the chemical changes due to the aromatic ring cross-linking reactions via condensation of the hydroxymethyl groups. Among the FTIR bands, the hydroxyl content and hydroxymethyl bands were among the regions of interest as an indication of cross-linking. As can be seen from Figure 3c, in both HL60NP and HL85NP, the intensity of the bands in the hydroxyl region at 3350 cm^–1^ decreased compared to the corresponding uncured samples. It is also clear that as a result of hydrothermal curing of HL85NP, the shoulder at 1290 cm^–1^ disappeared. These results unequivocally indicate that reactions consuming the hydroxymethyl groups occurred during the hydrothermal treatment.

To study the stability of cured HLNPs against dissolution in aqueous alkali, the basicity of samples was adjusted up to pH 13 by adding 1 M NaOH to the colloidal dispersions. Samples were stored at room temperature for 20 days and thereafter photographed and measured for particle diameter by DLS (Figure 3d). With increasing pH, the color tone of the colloids became darker due to the ionization of the phenolic hydroxyl groups (Figure S4). However, the systems remained colloidal as indicated by minor changes in their particle sizes. In addition, particles could be separated by centrifugation even from highly alkaline dispersions (Figure S5). Interestingly, after conditioning at pH 13, there was a slight increase in the particle diameters of both HLNP60 and HLNP85, indicating their swelling (Figure 3d). We chose to investigate the particles conditioned at pH 12 by SEM as the particle sizes remained unchanged and many base-catalyzed reactions can occur in this pH. As can be seen from the SEM images (Figure 3e,f), exposure to alkaline conditions did not visibly affect the shape and size of HLNPs. In addition to the alkaline stability of the cross-linked HLNPs, these particles showed stability in organic solvents such as ethanol, acetone, THF, and DMF (Figure S6), which presents opportunities for reactions in anhydrous organic solvents.

It has also been shown that during hydrothermal curing, carbon–carbon bonds can form and contribute to the formation of stable solvent particles at 190 °C.^51^ As a control experiment, we hydrothermally cured LNPs under the same conditions at 150 °C. Interestingly, these particles also showed good solvent and pH stabilities; however, they showed colloidal instability with time and aggregated, while the cured HLNPs did not show any sign of aggregation in 6 months (Figure S7). LNPs have previously been reported to have at most minor toxic effects on live cells^52,53^ and simple model organisms.^54^ Due to their resemblance to phenol–formaldehyde resins, we expect that the HLNPs developed in the present study are not themselves more toxic than regular LNPs; however, possible traces of free formaldehyde would present a point of concern. In accordance with the principle of precaution, we suggest that HLNPs are preferentially used in technical applications that do not involve biomedical or other live subjects. However, future study is anticipated to resolve this question of possible nanotoxicity and expand the possible end-use areas for these particles.

### 3.4 Self-Assembly of Silver Nanoparticles on the Surface of HLNPs

**Figure 4:**
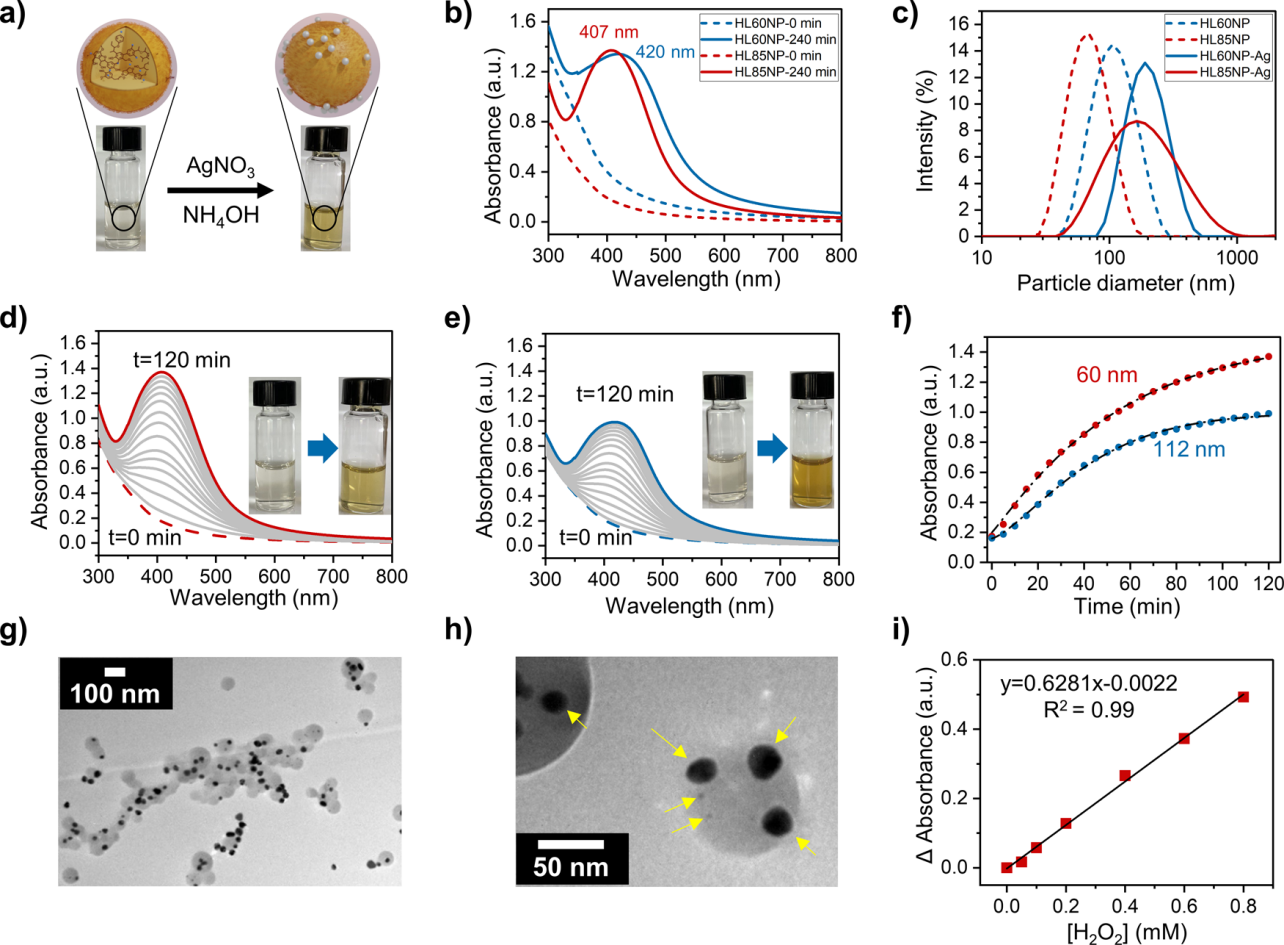
Self-assembly of silver nanoparticles on the surface of cross-linked HLNPs. (a) Scheme of formation of silver nanoparticles on the surface of LNPs. (b) UV–vis spectra of HLNPs before and after silver nanoparticles are assembled on the particle surface. (c) DLS particle size analysis of HLNPs before and after self-assembly of silver nanoparticles on the surface. (d) UV–vis spectra of silver nanoparticles formed on the HL85NP surface with a 60 nm diameter (data shown at 10 min intervals). (e) UV–vis spectra of silver nanoparticles formed on the HL85NP surface with a 110 nm diameter (data shown at 10 min intervals). (f) UV–vis kinetic plot of silver nanoparticle self-assembly on HL85NP with different particle sizes. (g) TEM image of HLNPs reacted with silver ammonia solution. (h) Silver nanoparticles formed on the surface of HLNPs with yellow arrows pointing on them. (i) Calibration curve for the detection of H_2_O_2_ using HLNP–silver hybrid nanoparticles.

It has been shown that addition of lignin into phenol–formaldehyde-based nanoparticles can be used for direct reduction of metallic ions and their surface-specific self-assembly.^26,55^ Wang et al. enzymatically cross-linked lignin for the synthesis of LNPs and thereafter silver–LNP hybrid particles. However, only the methanol-soluble fraction of lignin that was isolated with a low yield presented sufficient colloidal stability and spherical geometry for the reduction of the silver ions.^29^ Our starting point was HLNPs which were synthesized in an overall mass recovery yield of >75% and consist of ∼97% lignin based on the ^31^P NMR analysis (Figure 2). Taking advantage of their colloidal stability and preservation of the phenolic hydroxyl groups of lignin, we were curious to see whether it is possible to use HLNPs as a reducing agent and nucleation sites for silver ions under alkaline conditions. Addition of silver nitrate in ammonia solution to HLNPs resulted in an instantaneous color change toward a darker tone and upon dilution with water a goldish color appeared (Figure 4a). The cured particles with a smaller hydrodynamic radius (HL85NP, 60 nm) showed a faster color change compared to that achieved with larger particles. This color change was an indication of reduction of silver ions. Regardless of the particle size of cured HLNPs, there was a clear and time-dependent increase in the absorbance caused by the surface plasmon resonance (SPR) of silver at 407 nm for HL85NP and 420 nm for HL60NP (Figure 4b). To further elucidate the effect of the HLNP particle size on the size of final self-assembled silver nanoparticles, we studied the silver–lignin hybrid particles by DLS. The particle size distributions showed a clear increase in the hydrodynamic diameter upon silver reduction in HL60NP and HL85NP dispersions (Figure 4c). The broadening of the particle size distribution curves suggests that the presence of silver nanoparticles on the HLNPs make them less spherical, resulting in higher light scattering from such hybrid particles. Unlike the case of smaller particles (HL85NP, *d* = 60 nm), there was only minor broadening in the particle size distribution of larger particles (HL60NP, *d* = 112 nm). This difference indicates differences in reaction kinetics and surface coverage of the particles by metallic silver.

The formation and evolution of silver nanoparticles with time were studied using UV–vis spectroscopy. To limit the factors affecting the kinetics of nucleation, we used HLNPs prepared only from HL85 and modified the particle size by varying the initial concentration of HL85 and keeping the dispersion concentration of HL85NPs constant in the silver reduction reactions. The reactions were followed by the measurement of UV–vis absorbance spectra every 10 min for 2 h. There was a hypsochromic shift in the growing SPR to a lower wavelength as the particle size of HL85NPs decreased (Figure 4d,e). This can be attributed to the difference in the surface-to-volume ratios of the particles. Smaller nanoparticles have a higher surface area and hence there are a higher number of sites for heterogeneous nucleation of silver. Consequently, with a higher number of nuclei available, the silver ions available in the solution will be shared among higher number of seeds and there will be less free silver ions for the growth step. This results in a higher number of silver nanoparticles with a smaller size.

The kinetic plot of absorbance versus time shows how the rate of silver nanoparticle formation is higher in smaller particle sizes (Figure 4f). To test our hypothesis that the particle size of cured HLNPs directly affects the nucleation and growth of silver nanoparticles, the kinetic data were analyzed according to eq 1.
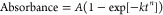
1

In this equation, the exponential factor *n* can be in the range of 1 to 4 and is equal to 1 in the case of ideal heterogeneous nucleation.^56^ By nonlinear curve fitting and minimization of least squares residuals, we were able to solve the Avrami parameters (*A*, *K*, and *n*). The results show that a more preferred heterogeneous nucleation behavior is evident in the case of HLNPs with a smaller particle size (HL85NP, *d* = 60 nm, *n* = 1.02) compared to the larger ones (HL85NP, *d* = 114 nm, *n* = 1.36). This finding supports our hypothesis that the particle size of cured HLNPs directly affects the formation of silver nanoparticles in the colloidal dispersion. It is thus possible to tune the surface coverage of the hybrid nanoparticles by using stabilized HLNPs of a predetermined size as the reducing agent.

To decipher the size and shape of the hybrid nanoparticles, and to find whether the silver nanoparticles are assembled on the surface and not just formed in the colloidal form in the dispersion, samples from the colloidal dispersion were taken for TEM imaging. As can be seen in Figure 4g,h, silver nanoparticles were almost exclusively observed on the surfaces of cured HLNPs. These results further indicate that the phenolic hydroxyl groups on the surface of cured HLNPs are the starting seeds of reduction of silver ions and thereafter the growth of these reduced silver nuclei is restricted on the surfaces. In fact, no formation of metallic silver was observed when a sample of HLNPs with etherified phenolic hydroxyl groups was used in the silver reduction step (data not shown).

Colloidally stable silver–lignin hybrid nanoparticles have many prospective applications such as sensory materials. Herein, we used Ag-HLNPs for the detection of H_2_O_2_ (Movie S1). These particles offer a facile means to quantify H_2_O_2_ with a concentration as low as 0.05 mM in the colloidal state using UV–vis spectroscopy (Figure 4i). There are not many prior studies combining lignin and silver nanoparticles for sensors. Among those, lignin has merely been used as a reducing and capping agent, that is, not as a spherical nanoparticle template. In addition, the synthetic procedures typically involve a low lignin concentration and a higher temperature compared to our system.^57,58^ Other studies have attempted to upscale the production of lignin-doped silver nanoparticles using an ionic liquid as a capping agent.^59^ Compared to more complicated and time-consuming synthesis, our procedure in an aqueous dispersion at room temperature requires only 3 h for completion. Furthermore, although our colloidal sensor could not challenge the low detection limit offered by electrochemical materials,^60−62^ our material is scalable and does not require conductive carbon materials. Although not studied in the present work, we anticipate that the hydrothermal curing of HLNPs renders them with a lower rate of biodegradability compared to their covalently unmodified counterparts. On the other hand, the redox activity of the cured HLNPs makes them an interesting topic for biodegradability studies with the possibility of enzyme-mediated formation of phenoxy radicals.^63,64^

### 3.5 Direct Chemical Modification of Cured HLNPs Using GTMA

**Figure 5:**
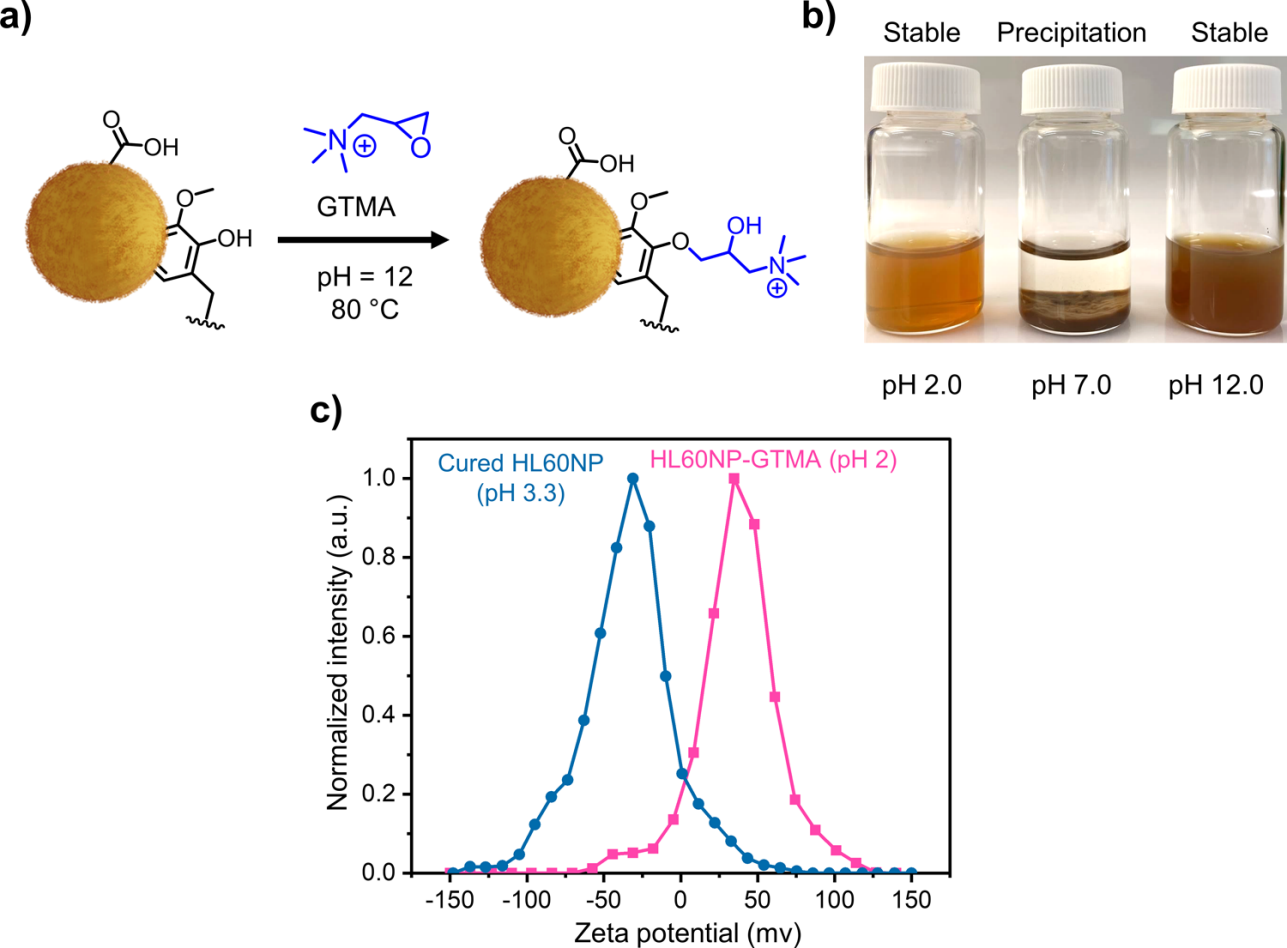
Chemical modification of self-cross-linked HLNPs. (a) Reaction scheme for ring-opening of GTMA on the surface of HLNPs as a nucleophilic agent. (b) pH-responsive colloidal behavior of HLNPs synthesized by the reaction of HL60NPs and GTMA. (c) Surface zeta potential of initial and modified HLNPs with GTMA.

The high pH resistance and structural stability of HLNPs make them a good candidate for direct covalent surface modification in the dispersion state. Here, we demonstrated amination of cured HL60NP by using the base-catalyzed oxirane ring-opening reaction (Figure 5a). In this reaction, GTMA reacts with the aliphatic and phenolic hydroxyl groups present on the surfaces of cured HLNPs.^34^ The presence of amino groups alongside hydroxyl groups with different p*K*_a_ values renders the particles with pH-dependent colloidal dispersibility (Figure 5b). It is worth noting that the modified particles will precipitate upon pH neutralization that occurs during dialysis against deionized water; however, upon decreasing or increasing the pH, they can be redispersed back to the colloidal state without any external modification such as sonication or manual shaking (Figure 5b). In contradiction to unmodified particles, the GTMA-modified particles show a positive zeta potential at pH 2 (+35 mV, Figure 5c) and do not coagulate at room temperature over an extended time duration (more than 1 month). It is also interesting to note that the quaternary ammonium groups rendered the particles with a narrower size distribution (Figure S8). This approach can open up a new window for targeting reactions at hydroxyl groups in the colloidal state instead of chemical modifications before particle formation that could adversely affect the self-assembly and stability of the nanoparticles.

## 4 Conclusions

We have prepared internally cross-linked nanoparticles from HL via facile solvent shifting precipitation and hydrothermal curing. Such a scalable approach not only preserves the phenolic hydroxyl groups of lignin but also improves the reactivity and stability of the nanoparticles under synthetically relevant conditions. We showed that the stabilized particles function as reducing agents for silver ions to give rise to silver–lignin hybrid particles that function as facile colloidal sensors for H_2_O_2_. The stability under alkaline conditions can also be exploited for base-catalyzed etherification in the colloidal state as we demonstrated with the oxirane ring-opening reaction with GTMA. These modified particles show pH-responsive reversible agglomeration and redispersion properties. Both of these chemical modifications offer promising paths toward antimicrobial materials, and we anticipate that it would also be possible to combine the two functionalities to synthesize cationic silver–LNPs. Overall, the stabilized LNPs presented herein open up new opportunities for versatile chemistry and pave the way for circular biobased materials.

## Supporting Information Available

^31^P NMR analysis of SKL, HL60, and HL85; effect of concentration on the HLNP particle size; DSC analysis of curing of HL60 and HL85; solubility comparison of SKL, HL60, and HL85 after curing; alkaline and organic solvent stabilities of cured HL60NP and HL85NP; LNP and HLNP curing behaviors; and size analysis of GTMA-modified HLNP using DLS (PDF)
Application of the Ag-HLNP system for the detection of H_2_O_2_ (MP4)
