# Supplementary material for: Stabilized Lignin Nanoparticles for Versatile Hybrid and Functional Nanomaterials
Source: Biomacromolecules. 2022 Oct 14;23(11):4597–606. doi: 10.1021/acs.biomac.2c00840 (PMC9667459; doi:10.1021/acs.biomac.2c00840)
Supplement: Supplementary file 1 — bm2c00840_si_001.pdf [file bm2c00840_si_001.pdf]

Supporting information for:

Stabilized lignin nanoparticles for versatile hybrid and functional nanomaterials

Mohammad Morsali[1], *Adrian Moreno*[1,2], *Andriana Loukovitou*[1, 3], *Ievgen Pylypchuk*[1], *Mika H. Sipponen*[1,*]

M. Morsali, Dr. A. Moreno, A. Loukovitou, Dr. I. Pylypchuk, Prof. Dr. M. H. Sipponen

1- Department of Materials and Environmental Chemistry, Stockholm University, Svante Arrhenius väg 16C, SE-106 91, Stockholm, Sweden

E-mail: mika.sipponen@mmk.su.se

2- Current address: Laboratory of Sustainable Polymers, Department of Analytical Chemistry and Organic Chemistry, University Rovira i Virgili, Tarragona 43007, Spain

3- Current address: Faculty of Chemistry and Biochemistry, Ruhr University Bochum, Universitätsstr. 150, 44780 Bochum, Germany

This PDF contains:

Table S1

Figures S1—S8

Caption for Movie S1

Table S1. ^{31}P NMR analysis of kraft lignin and hydroxymethylated lignin.

Sample	Aliphatic	G-cond-1	S or G-cond-1	S or G-cond-2	G-nc	H	COOH	Tot. Phenolic	Total OH
**Kraft lignin**	1.96	0.13	1.63	0.88	1.24	0.35	0.53	4.23	6.73
**HL60**	2.70	0.13	2.29	0.97	0.99	0.20	0.46	4.59	7.75
**HL85**	3.26	0.14	2.67	1.03	0.35	0.10	0.43	4.29	7.99

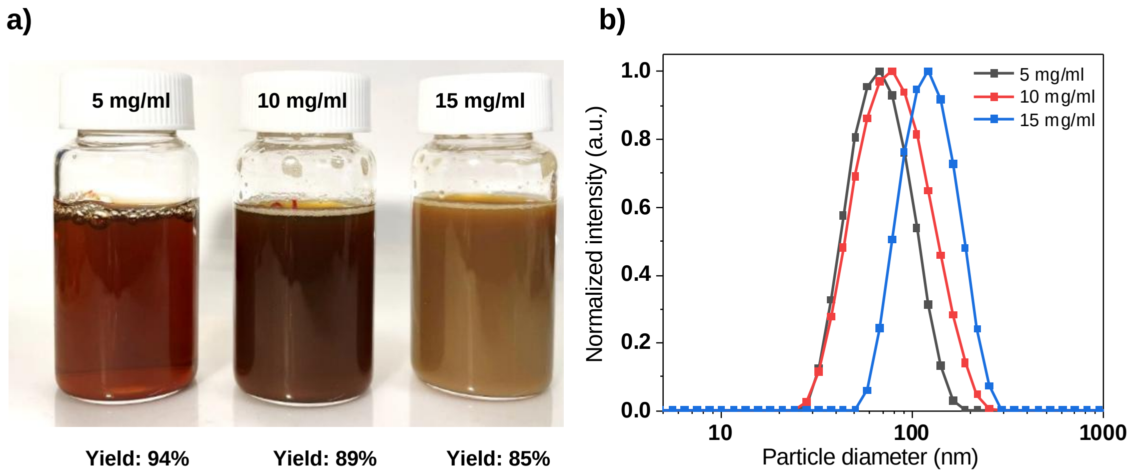

Figure S1. Effect of initial hydroxymethylated lignin concentration on final particle size. a) Digital image of HLNPs with different final concentrations (from left to right: HL85NP with 5 mg/ml , 10 mg/ml and 15 mg/ml lignin). b) Particle size distribution (DLS, hydrodynamic diameter) of HLNPs. (yield determines the final concentration of hydrocolloids compared to nominal concentration calculated).

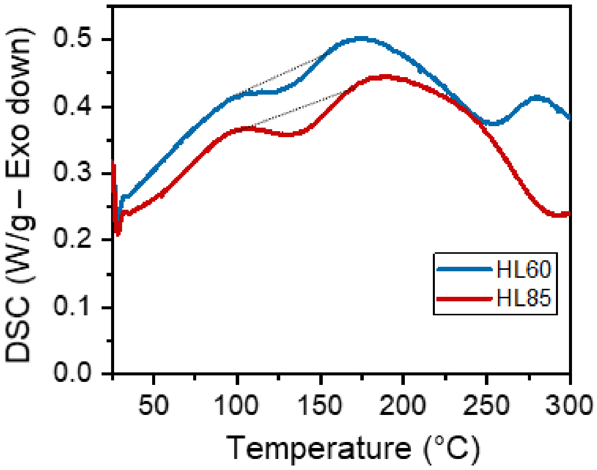

Figure S2. Differential scanning calorimetry (DSC) analysis of lignin hydroxymethylated at 60 °C (HL60) and 85 °C (HL85).

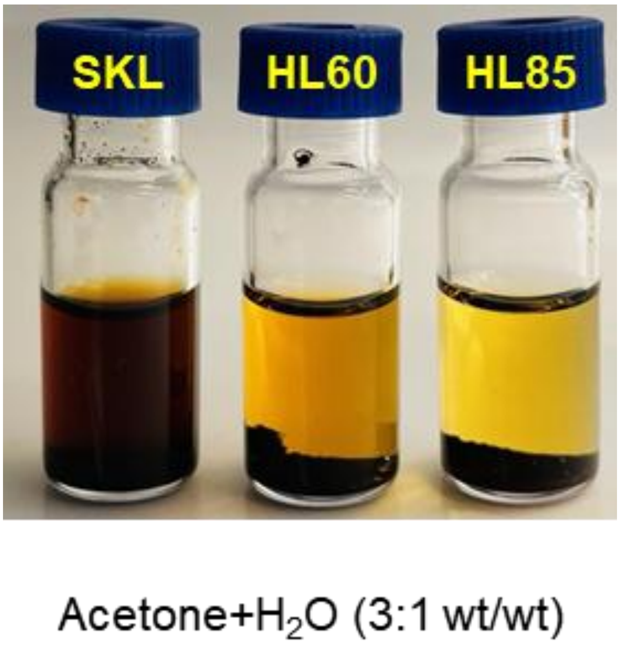

Figure S3. Visual comparison of solubility of SKL, HL60 and HL85 after thermal treatment at 150 °C overnight. (SKL: softwood Kraft lignin, HL60: lignin hydroxymethylated at 60 °C, and HL85: lignin hydroxymethylated at 85 °C)

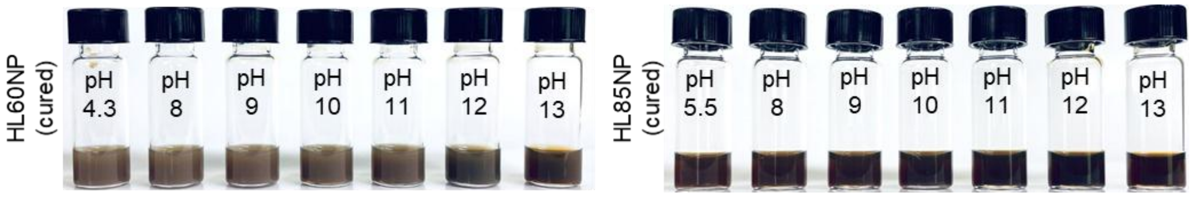

Figure S4. Stability of cured HLNPs in alkaline aqueous solutions at different pH.

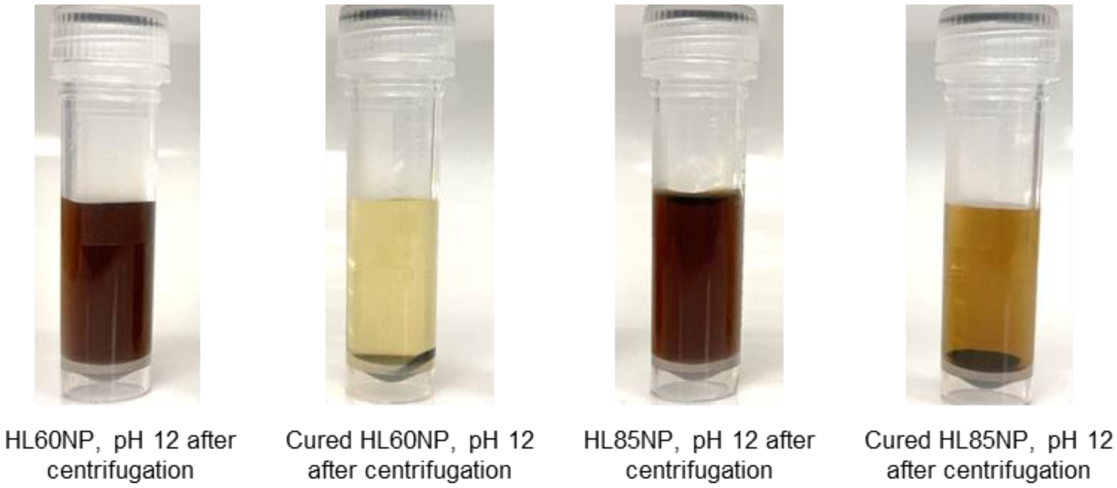

Figure S5. Effect of curing on alkaline stability of cured HL60NP and HL85NP. Comparison of stability of cured and cured particles in pH12 using centrifugation. Please note that the particles will separate during centrifugation if they are insoluble while in the case of uncured particles, they form a solution which cannot be separated by centrifugation. (HL60NP: nanoparticles from hydroxymethylated lignin at 60 °C, HL85NP: nanoparticles from hydroxymethylated at 85 °C)

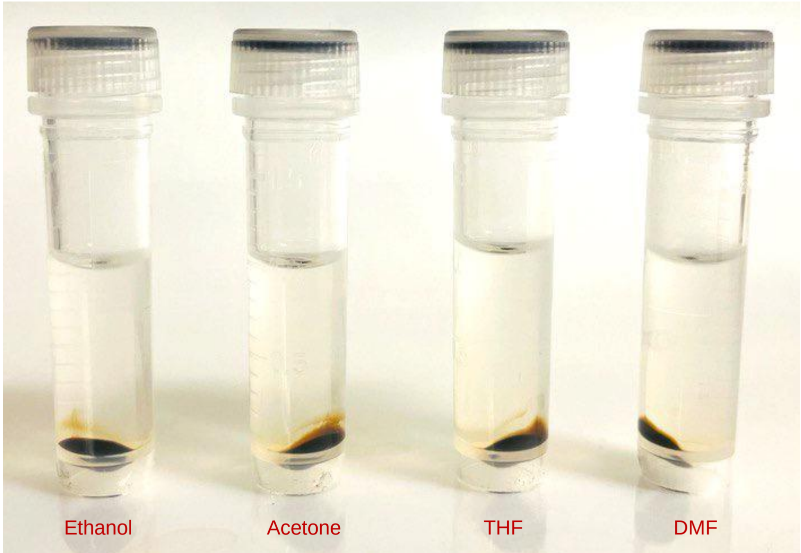

Figure S6. Solvent stability of cured HL85NP with 100 nm diameter in different solvents demonstrated using extensive centrifugation. Please note that particles are separated after centrifugation to demonstrate their insolubility. (HL85NP: nanoparticles from lignin hydroxymethylated at 85 °C).

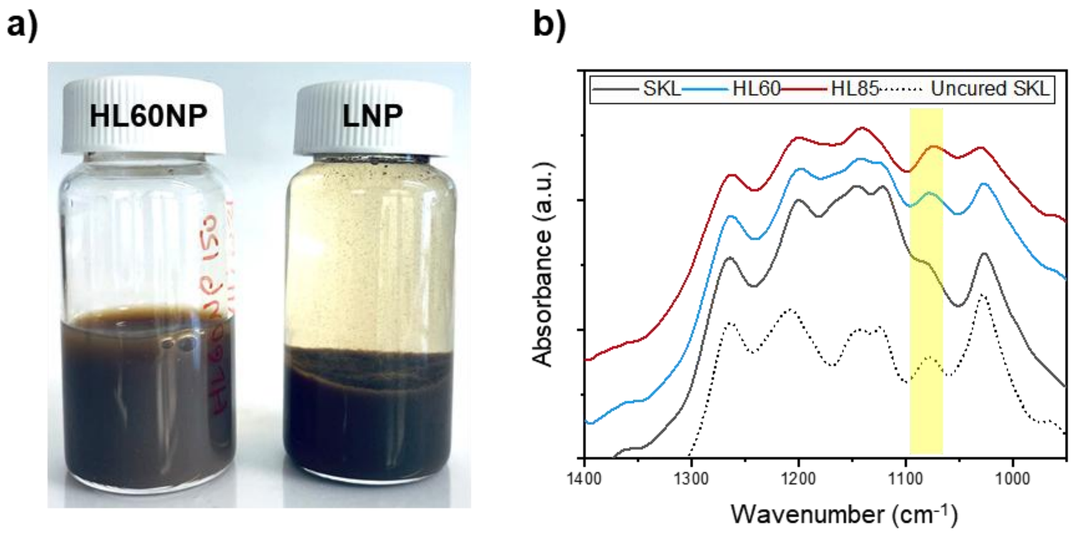

Figure S7. Effect of hydroxymethylation on thermal treating of LNPs. a) Colloidal stabilty of cured HL60NP after 6 months (left) compared to cured LNP (right) after 3 months. b) FTIR spectrum of SKL, HL60 and HL85 after thermal treatment. (SKL: softwood Kraft lignin, HL60: lignin hydroxymethylated at 60 °C, and HL85: lignin hydroxymethylated at 85 °C)

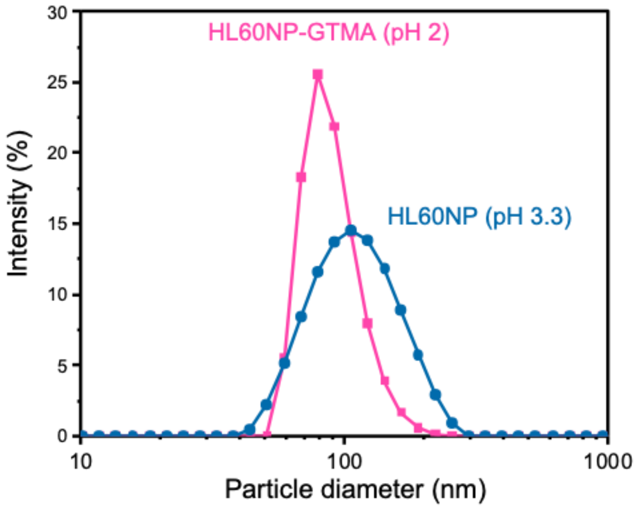

Figure S8. Size measurement analysis using DLS of modified and unmodified HLNPs with GTMA.

Supporting Movies:

Movie S1. The procedure for implementing Ag-HLNP system for detection of hydrogen peroxide (concentration = 1 mM) present in the system. (Video is cropped before addition of hydrogen peroxide, but the video playback speed is in real time)

To 2 mL phosphate buffer (20 mM, pH 7.4), were added 10 µL of Ag-HLNP stock colloid. After mixing and homogenizing the sample using a micropipette. Then required amount of hydrogen peroxide (from stock solution with 20 mM concentration) is added.
